# Supplementary material for: Communication is key: Mother-offspring signaling can affect behavioral responses and offspring survival in feral horses (Equus caballus)
Source: PLoS One. 2020 Apr 17;15(4):e0231343. doi: 10.1371/journal.pone.0231343 (PMC7164835; doi:10.1371/journal.pone.0231343)
Supplement: S1 Table — (-) indicate that mares were not observed in a particular year; (&) indicate double male bands; (*) indicate changes in band stallion within that year of observation. (DOCX) [file pone.0231343.s001.docx]

**S1 Table. Individual study subject details.**

|  |  | **Year** |  |
| --- | --- | --- | --- |
| **Mare** | **1995** | **1996** | **1997** |
| Annie | Jason & Adonis | - | - |
| Arp | - | - | Spock |
| Bandit | - | Lucifer  *Perseus | - |
| Cathy | - | - | Adam |
| Daphne | Excel  *Lightfoot | Lightfoot | - |
| Dawn | - | Euclid | - |
| Delta | Stegasus | Stegasus | Satellite |
| Doobie | Dionysus | Dionysus | - |
| Dot | - | Stegasus | - |
| Electra | Stegasus | Stegasus | Satellite |
| E’s C | Lightfoot | Lightfoot | - |
| Eve | - | Dionysus | - |
| Holly | - | - | Master |
| Horace | - | Mitchell & Medusa  *Mitchell | Adam |
| Jennifer | Mitchell & Medusa | Mitchell & Medusa | Medusa |
| June | - | Solomon | - |
| Leila | Jason & Adonis | Lenon | Lenon |
| Luna | Caesar | - | Spock |
| Moonshadow | Jason & Adonis | - | - |
| Nadia | - | Euclid | - |
| Nina | - | Stegasus | - |
| Sapphire | Woden | Woden | Spock |
| Sarah | - | Lenon | - |
| Scar | - | Adam  *Zane | Adam |
| Siren | Woden | Woden | Winston |
| Slug | - | Dionysus  *Lightfoot | Dionysus |
| Solvi | - | Caesar | - |
| Spalding | Zane | Zane | Dino |
| Spot | Lightfoot | - | - |
| Squiggs | Dionysus | Dionysus | - |
| Texas | - | Lightfoot | Dionysus |
| Triscuit | Case | Case | Lizard |
| Tuna | - | BS & Stanley  * Stanley | Stanley |

(-) indicate that mares were not observed in a particular year; (&) indicate multi-stallion bands;

(*) indicate changes in band stallion within that year of observation.
