# Supplementary material for: Communication is key: Mother-offspring signaling can affect behavioral responses and offspring survival in feral horses (Equus caballus)
Source: PLoS One. 2020 Apr 17;15(4):e0231343. doi: 10.1371/journal.pone.0231343 (PMC7164835; doi:10.1371/journal.pone.0231343)
Supplement: S2 Table — (DOCX) [file pone.0231343.s002.docx]

**S2 Table. AICc analysis for models with and without band as a random factor.**

| **Test** | **Model** | **AICc with band** | **AICc without band** |
| --- | --- | --- | --- |
| **GLMM** | Mare vs. foal initiation | 794.0493 | **783.9917** |
|  | Snort use probability | 528.2413 | **528.335** |
|  | Nicker use probability | 419.1914 | **419.0543** |
|  | Whinny use probability | **457.8675** | 461.2089 |
|  | Communication outcomes | 622.1653 | **620.3465** |
| **LME** | Mare and foal communication rate comparison | **638.7177** | 643.7331 |
|  | Mare and foal communication rate correlation | **1373.403** | 1409.518 |
|  | Mare communication initiation rate | **698.9061** | 705.8789 |
|  | Foal communication initiation rate | **654.7780** | 657.7344 |
