# Supplementary material for: Communication is key: Mother-offspring signaling can affect behavioral responses and offspring survival in feral horses (Equus caballus)
Source: PLoS One. 2020 Apr 17;15(4):e0231343. doi: 10.1371/journal.pone.0231343 (PMC7164835; doi:10.1371/journal.pone.0231343)
Supplement: S3 Table — (DOCX) [file pone.0231343.s003.docx]

**S3 Table. Model output for insignificant main effects.**

| **Test** | **Model** | **Variable** | **Estimate** | **SE** | **Test statistic** | ***P*-value** |
| --- | --- | --- | --- | --- | --- | --- |
| **GLMM** | Mare vs. foal initiation | Habitat visibility | 0.02 | 0.22 | *z* = 0.09 | 0.92 |
|  |  | Foal sex | 0.38 | 0.36 | *z* = 1.05 | 0.29 |
|  |  | Mare age | -0.03 | 0.05 | *z* = -0.57 | 0.56 |
|  | Snort use probability | Foal sex | 0.17 | 0.34 | *z* = 0.52 | 0.60 |
|  |  | Mare age | -0.01 | 0.05 | *z* = -0.33 | 0.74 |
|  | Nicker use probability | Habitat visibility | 0.07 | 0.31 | *z* = 0.22 | 0.82 |
|  |  | Foal sex | -0.11 | 0.41 | *z* = 0.26 | 0.80 |
|  |  | Foal age | -0.02 | 0.01 | *z* = -1.51 | 0.13 |
|  |  | Mare age | -0.04 | 0.05 | *z* = -0.80 | 0.42 |
|  | Whinny use probability | Foal sex | -0.39 | 0.42 | *z* = -0.93 | 0.35 |
|  |  | Mare age | 0.04 | 0.06 | *z* = 0.75 | 0.45 |
| **LME** | Mare and foal communication initiation correlation | Foal sex | -0.02 | 0.05 | *t* = -0.36 | 0.74 |
|  |  | Foal age | 0.002 | 0.001 | *t* = 1.28 | 0.20 |
|  |  | Mare age | 0.007 | 0.006 | *t* = 1.15 | 0.25 |
|  | Mare communication initiation rate | Foal sex | 0.10 | 0.07 | *t* = 1.49 | 0.21 |
|  |  | Foal age | 0.0007 | 0.002 | *t* = 0.43 | 0.70 |
|  |  | Mare age | -0.01 | 0.009 | *t* = -1.36 | 0.17 |
|  | Foal communication initiation rate | Foal sex | 0.01 | 0.05 | *t* = 0.24 | 0.81 |
|  |  | Foal age | 0.001 | 0.001 | *t* = 0.91 | 0.36 |
|  |  | Mare age | 0.003 | 0.006 | *t* = 0.40 | 0.70 |
| **Survreg** | Foal survival and behavior at 0-10 weeks | Average suckling rate | 0.14 | 0.12 | *z* = 1.23 | 0.21 |
|  |  | Average mare-foal distance | 0.01 | 0.01 | *z* = 1.12 | 0.26 |
|  |  | Foal sex | -0.04 | 0.18 | *z* = -0.19 | 0.84 |
|  |  | Mare age | 0.008 | 0.02 | *z* = 0.31 | 0.60 |
|  | Foal survival and behavior at 10-20 weeks | Average suckling rate | -0.04 | 0.18 | *z* = -0.25 | 0.75 |
|  |  | Average mare-foal distance | 0.008 | 0.01 | *z* = 0.78 | 0.43 |
|  |  | Mare age | -0.007 | 0.02 | *z* = -0.40 | 0.70 |
