# Supplementary material for: Communication is key: Mother-offspring signaling can affect behavioral responses and offspring survival in feral horses (Equus caballus)
Source: PLoS One. 2020 Apr 17;15(4):e0231343. doi: 10.1371/journal.pone.0231343 (PMC7164835; doi:10.1371/journal.pone.0231343)
Supplement: S4 Table — (DOCX) [file pone.0231343.s004.docx]

**S4 Table. Models separately estimating the effects of initiator type and signal use on the likelihood of suckling, distance decrease, and no-change outcomes.**

| **Outcome** | **Interaction with initiator type** | **Signal used** | **Estimate** | **SE** | ***z* - score** | ***P* - value** |
| --- | --- | --- | --- | --- | --- | --- |
| Suckling | *P* = 0.84 | Snort | -3.48 | 0.42 | -8.22 | < 0.0001 |
|  | *P* = 0.62 | Nicker | 2.32 | 0.43 | 5.41 | < 0.0001 |
|  | *P* = 0.98 | Whinny | 2.05 | 0.39 | 5.28 | < 0.0001 |
| Distance decrease | *P* = 0.11 | Snort | -2.19 | 0.26 | -8.32 | < 0.0001 |
|  | *P* = 0.20 | Nicker | 1.22 | 0.31 | 3.90 | < 0.0001 |
|  | *P* = 0.47 | Whinny | 1.28 | 0.25 | 5.04 | < 0.0001 |
| No-change | *P* = 0.07 | Snort | 1.90 | 0.29 | 6.51 | < 0.0001 |
|  | ***P* = 0.05** | Nicker (foal) | -1.78 | 0.39 | -4.55 | < 0.0001 |
|  |  | Nicker (mare) | 1.07 | 0.55 | 1.96 | 0.05 |
|  | *P* = 0.58 | Whinny | -1.41 | 0.30 | -4.71 | < 0.0001 |
